# Multicompartmental analysis of microbiome alterations under radiation stress

**DOI:** 10.1186/s13568-025-02002-4

**Published:** 2025-12-31

**Authors:** Nurislam Mukhanbetzhanov, Sanzhar Zhetkenev, Elizaveta Vinogradova, Shynggys Sergazy, Zharkyn Jarmukhanov, Argul Issilbayeva, Alibek Kossumov, Aliya Kassenova, Bagdaulet Mukhametuly, Murat Kenessarin, Kassym Zhumadilov, Valeriy Stepanenko, Viktorya Bogacheva, Aleksei Petukhov, Hitoshi Sato, Satoru Endo, Nariaki Fujimoto, Masaharu Hoshi

**Affiliations:** 1 National Laboratory Astana, Center for Life Sciences, Nazarbayev University, Z05H0P9 Astana, Republic of Kazakhstan; 2 Institute of Nuclear Physics of ME RK, 050032 Almaty, Republic of Kazakhstan; 3 A. Tsyb Medical Radiological Research Center - Branch of the National Medical Research Radiological Centre of the Ministry of Health of the Russian Federation, 4 Koroleva St., Obninsk, Kaluga Region Russian Federation 249036; 4 Ibaraki Prefectural University of Health Sciences, 4669-2 Ami, Ami-machi, Inashiki-gun, Ibaraki 300-0394 Japan; 5 Research Institute for Radiation Biology and Medicine, Hiroshima University, 1-2-3, Kasumi, Minami-ku, Hiroshima, 734-8551 Japan; 6 The Center for Peace, Hiroshima University, Higashisenda-machi 1-1-89, Naka-ku, Hiroshima, 730-0053 Japan; 7 International Department of Nuclear Physics, New Materials and Technology, L. N. Gumilyov Eurasian National University, 13 Munaitpasova St., 010008 Astana, Republic of Kazakhstan; 8 Graduate School of Advanced Science and Engineering, Hiroshima University, 1-4-1, Kagamiyama, Higashi, Hiroshima, 739-8527 Japan

**Keywords:** Gut microbiota, Silicon dioxide, Irradiation, Nanoparticles, Radioactive, Exposure

## Abstract

Radioactive compounds, including silicon dioxide (^31^SiO_2_), may enter the environment and affect biological systems, yet their impact on the gut microbiota remains poorly studied. This study aimed to evaluate the effects of radioactive ^31^SiO_2_, compared to non-radioactive SiO_2_ and gamma irradiation, on the composition and functionality of the gut microbiota in laboratory Wistar rats (n = 28). Gut microbiota analysis of the large intestine, small intestine, and Peyer’s patches was conducted at 90 min and 72 h post-exposure using 16S rRNA sequencing. Alpha-diversity results showed increased species richness in Peyer’s patches at 90 min after exposure to radioactive ^31^SiO_2_, with elevated *Bacilli* and Gammaproteobacteria, a trend persisting at 72 h. In the small intestine, radioactive ^31^SiO_2_ increased *Bacilli* and *Clostridia* at 3 days, while Gammaproteobacteria considerably decreased; by 72 h, Bacilli and *Clostridia* maintained growth, with *Gammaproteobacteria* recovering. These findings demonstrate that radioactive ^31^SiO_2_ dynamically alters gut microbiota composition and diversity, highlighting its unique influence on microbial communities.

**Supplementary Information:**

The online version contains supplementary material available at 10.1186/s13568-025-02002-4.

## Introduction

The intricate relationship between environmental exposures and gut microbiota perturbations represents a frontier in understanding radiation-induced pathophysiology. While various environmental factors—including diet, xenobiotics, and pathogens—are known to modulate gut microbiota, the impact of radioactive compounds, particularly those commonly encountered in industrial and medical settings, remains poorly understood (Carding et al. 2015). In addition to the radiation present in nature, all organisms are exposed to alpha, beta, and gamma rays. Beta radiation, consisting of electrons or positrons, is inferior in strength to alpha radiation, but has a much longer range. Gamma rays, due to their high penetrating ability, can pass freely through the entire human body, affecting deep tissues. Gamma radiation is widely used in medicine: sublethal doses from 6 to 10 Gy are effective in bone marrow transplantation, immune suppression, and cancer therapy. Although such treatment is an important tool in clinical practice, it carries serious risks for the body. The gastrointestinal tract, which is sensitive to the effects of such doses, is particularly vulnerable. Radiation has a noticeable effect on the cells of the intestinal epithelium and the microflora inhabiting the mucous membrane of this organ (Sittipo et al. 2020).

Irradiation with a dose of at least 6 Gy for one hour can cause several serious consequences for the human body, such as severe nausea, vomiting and diarrhea, which, in turn, can lead to severe dehydration of the body. Over the next 4–5 days, the human condition improves, but significant damage occurs in the gastrointestinal tract, the cells of the mucous membrane of the digestive tract, which usually serve as a protective barrier, begin to collapse and die (Talapko et al. 2024).

Among the radioactive compounds, silicon dioxide SiO_2_, especially its activated isotope ^31^SiO_2_, stands out due to its wide application and potential for interaction with biological systems.

Silicon dioxide (^31^SiO_2_), ubiquitous in modern technology and consumer products, emerges as a crucial model for investigating radiation-microbiome interactions, especially when considering its neutron-activated isotope, ^31^SiO_2_ (Diao et al. 2021).

The unique physicochemical properties of silica nanoparticles (SiNPs), including their uniform pore size, controllable particle dimensions, extensive surface area, and readily modifiable silanol groups (Si–OH), have established them as versatile platforms in numerous applications. Their exceptional biocompatibility, coupled with their widespread presence in construction materials, microelectronics, and food additives, raises critical questions about their potential impact on biological systems, particularly when radioactively activated (Huang et al. 2022; Diao et al. 2021; Yang et al. 2016; Younes et al. 2018).

Recent evidence suggests that even non-radioactive ^30^SiO_2_ nanoparticles can significantly alter gut microbiota communities. Preliminary studies using human-equivalent doses (2.5 mg/kg body weight daily) demonstrated unexpected increases in microbial diversity and richness after just one week of exposure. However, these findings represent only a fraction of the potential biological implications, particularly when considering radioactive variants (Fruijtier-Pölloth 2016).

The interaction between radiation and the gut microbiota presents a complex paradigm with far-reaching consequences for human health. Recent studies shed light on the role of the gut microbiota in radiation exposure, which resulted in a change in the bacterial composition in the gut microbiota, where the number of potential harmful bacteria increased and beneficial decreased, which can lead to an inflammatory reaction and damage to the intestinal mucosa. (Lu et al. 2024). This mechanism may represent a critical, yet underexplored, component of radiation injury pathogenesis.

The novelty of our investigation lies in comparative examining the impact of neutron-activated silicon dioxide (^31^SiO_2_) microparticles, gamma irradiation and non-activated silicon dioxide (^30^SiO_2_) microparticles on gut microbiota—a unique approach that bridges the gaps between radiation biology, materials science, and gut microbiota research.

This study specifically addresses three critical knowledge gaps, first is the temporal dynamics of gut microbiota responses to radioactive compounds, for this reason, in our study, animal euthanasia was performed for 90 min due to the fact that it allows us to identify the body’s immediate reactions to radiation and 72 h allows us to study the long-term effect after exposure, similar to the earlier study conducted by (Abishev et al. 2023) studied the effects of ^56^MnO_2_ microparticles on the lungs of C57BL mice, where euthanasia was performed at certain time intervals to assess the radiation effects. The second is compartment-specific alterations of microbial communities in small intestine, large intestine, fecal and Peyer’s patches following radiation exposure and the identification of potential gut microbiota biomarkers for radiation injury.

This research not only advances our fundamental understanding of radiation-microbiome interactions but also opens new avenues for developing gut microbiota-based interventions for radiation injury.

## Materials and methods

All experimental protocols and animal care procedures were conducted in accordance with ARRIVE guidelines (Percie du Sert et al. 2020) and EU Directive 2010/63/EU on the protection of animals used for scientific purposes. The study received ethical approval from the Ethics Committee of Kazakh National Medical University named after S.D. Asfendiyarov (Protocol #7 (145), September 7, 2023, Almaty, Republic of Kazakhstan). Animals were maintained in vivarium conditions with ad libitum access to standard chow and tap water. Environmental parameters were controlled at 19–22 °C ambient temperature, 30–70% relative humidity, and a 12-h light–dark photo period. Animal weights were recorded three days prior to experimental procedures.

### Study design

**Fig. 1:**
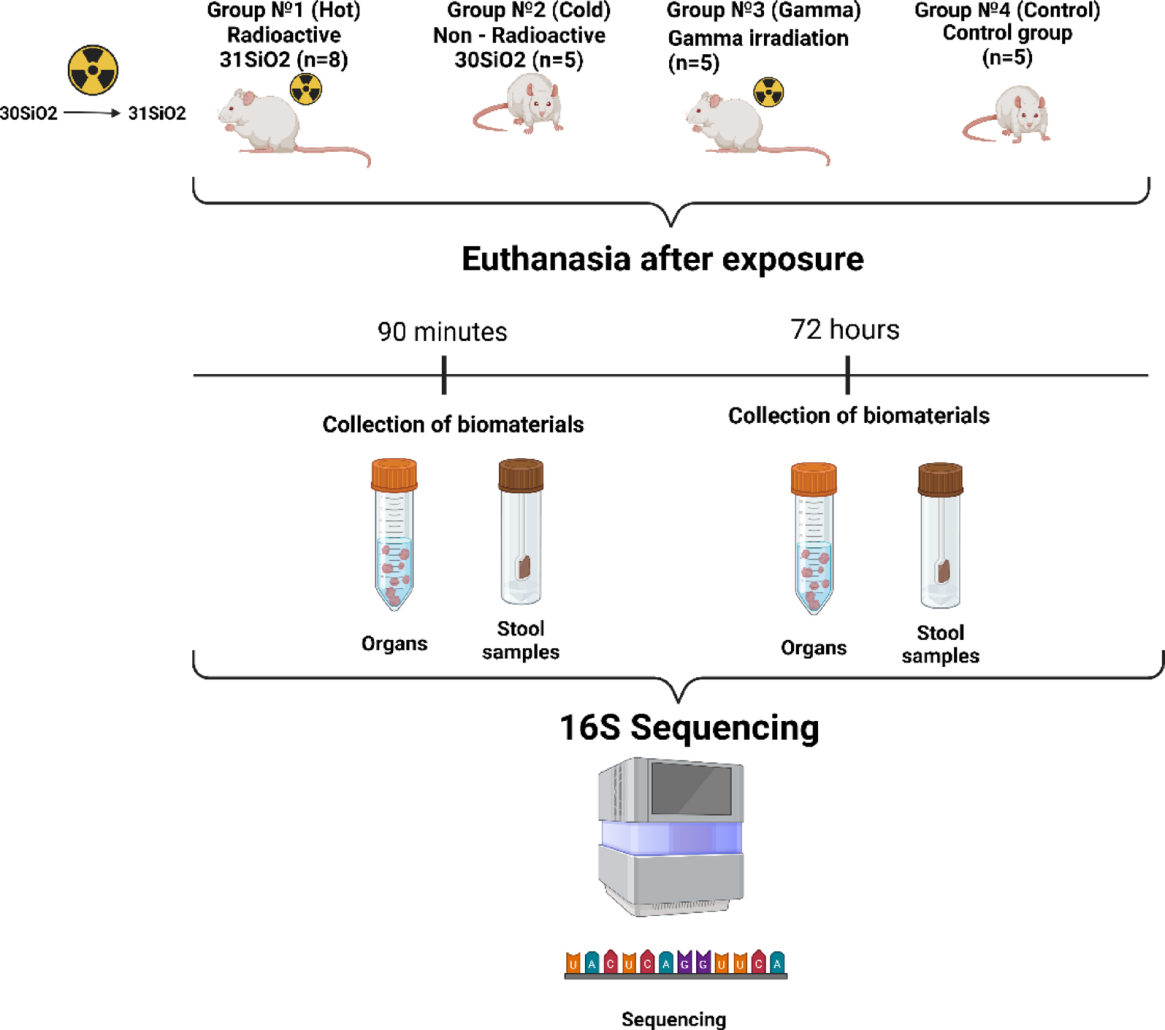
Study design

The study used 23 ten-week-old male Wistar rats (outbred, specific pathogen-free status) with an average weight of 310 g ± 73.27 g. These animals were kept in plastic cages under controlled conditions including a 12-h light/dark cycle and temperature maintained at 23 °C ± 2 °C. The rats were divided into four distinct groups (illustrated in Fig. 1, generated using https://BioRender.com).

- Hot group (^31^SiO_2_) (n = 8) was exposed to sprayed neutron-activated powder of ^31^SiO_2_ microparticles (radioactive microparticles). In total, three rats from the first ^31^SiO_2_ group were euthanized for dosimetry 90 min after hour exposure.
- Cold group (^30^SiO_2_) (n = 5) was exposed to sprayed exposure to non-radioactive powder of ^30^SiO_2_ microparticles (non-radioactive silicon dioxide microparticles).
- Gamma group (n = 5) was receiving single whole-body gamma radiation exposure.
- Control group (n = 5) was control group, did not undergo intervention.

In total, 3 out of 8 rats from the first group (Hot ^31^SiO_2_ microparticles) were euthanized for dosimetry 90 min after exposure. The remaining 5 rats were euthanized 72 h after exposure. Rats from the second group (Cold ^30^SiO_2_) were exposed by non-radioactive silicon dioxide microparticles (Cold ^30^SiO_2_). Animals in the third group (Gamma) were receiving single whole gamma radiation exposure. The last group was a control group, which was not undergoing any intervention. All organ and stool samples in the control group were collected 72 h after the start of the study, which corresponded to the time of euthanasia in the groups with Cold (^30^SiO_2_), Gamma and remaining Hot (^31^SiO_2_). The 90-min time interval was not included for the cold ^30^SiO_2_ group, since at this early stage the main task was to study the effect of radioactive particles of silicon dioxide ^31^SiO_2_, which required immediate dosimetry. It was also expected that non-radioactive silicon dioxide microparticles (Cold ^30^SiO_2_) would cause minimal changes in the gut microbiota within 90 min, as its effects would probably manifest over a longer period. Rats were placed in a desiccator with cotton soaked in isoflurane (Isoflurane; Fujifilm Wako Pure Chemical Corporation, Japan) for anesthesia, followed by euthanasia and blood collection from abdominal artery. Large intestine (LI), small intestine (SI), fecal samples (F) and Peyer’s patches (P) all these organs were dissected, extracted from each animal by group and were weighed, and preserved in 10% neutral buffered formalin (NBF), then stored in refrigerator at + 4 °C next days.

### Silicon dioxide microparticles activation and radiation measurements

^31^SiO_2_—silicon dioxide powder was obtained from Admatechs (Shanghai) Co., Ltd. (Shanghai, China), with particles diameters ranging from 0.2 to 2.0 μm. Activation of ^31^SiO_2_ was achieved through neutron activation using WWR-K nuclear research reactor with low-enriched fuel at the “Institute of Nuclear Physics” in Almaty, Kazakhstan by thermal neutrons flux of 7.26 × 10^13^ neutron/(cm^2^ × s) with duration of irradiation equal to 300 s. The activity of ^31^Si (T_1/2_ = 157.3 min) obtained as a result of neutron activation was equal to 4.92 × 10^7^ Bq per 1 g of ^30^SiO_2_ microparticles.

For the experiment, a specialized isolation chamber equipped with forced ventilation and an air filtration system was employed to maintain animal respiration. A pneumatic system distributed radioactive powder to the animals within the chamber via airflow, as described in our previous study (Stepanenko et al. 2024).

1.5 g of ^31^SiO_2_ powder placed in a U-shaped tube. A 9 mm U-shaped tube was positioned at the connection point in the spray channel. The low-pressure breathing channel was closed, and the high-pressure spray valve was opened 2–3 times to spray radioactive powder before closing. Low-pressure breathing channel remained open for 3 h.

Activated ^31^SiO_2_ powder sprayed in sealed cells. One hour after the experiment, rats were removed from exposure boxes, placed in new cells for 0.5 h to cool, and then euthanized. Samples from each organ were prepared and weighed. Radioactivity of ^31^SiO_2_ in each sample was measured using NaI detector and GM counter as described by (Stepanenko et al. 2024). Absorbed doses of internal radiation were estimated using data on ^31^SiO_2_ activity measurements with accounting for values of “Absorbed energy fractions” (AF) for beta-particles and gamma-quanta irradiated by ^31^Si in considered organ and tissues of experimental rats. Values of AF were calculated using the Monte Carlo code (MCNP-4C version) and rat’s mathematical phantom. For the experiment to spray non-radioactive ^30^SiO_2_ powder was used the separate box, rats were sprayed with 1.5 mg of ^30^SiO_2_ powder without neutron activation.

### Sample collection and storage

All experiments were conducted under fully sterile conditions, with dissection tools changed for each animal to prevent cross-contamination. Organs (including Peyer’s patches) for microbiome analysis were immediately frozen and stored at − 80 °C. Samples of small intestine (SI) n = 20, large intestine (LI) n = 22, fecal (F) n = 20, Peyer’s patches (P) n = 23, were collected from all animals for histological examination and stored in 10% neutral buffered formalin (NBF), then stored in refrigerator at + 4 °C next days. Fecal collection involved placing 100–250 mg of fresh fecal in sterile 2 ml tube, which were immediately frozen and stored at − 30 °C until DNA extraction process begins.

### DNA extraction, quality control and sequencing

DNA extraction from all samples is performed in a dedicated sterile laminar flow hood using dedicated pipettes and consumables. Genomic DNA from fecal and organ samples was extracted using the ZymoBIOMICS DNA Miniprep Kit (Zymo Research, D4300), and sterile µQ water was used as a negative extraction control. OD260/280 Nanodrop and electrophoresis performed qualitative control of DNA isolation in a 1% agarose gel. The concentration and purity of each DNA sample were determined using an Invitrogen Qubit 3.0 Fluorimeter (Invitrogen, Carlsbad, California, United States). Sterile µQ water served as a negative control. Following the standard Illumina protocols, the samples were sequenced at Novogene (Beijing, China) on the Illumina NovaSeq 6000 platform. 16S rRNA sequencing targeted the V3-V4 hypervariable regions, which provides robust taxonomic resolution for intestinal microbiota analysis.

### Bioinformatic and statistical analysis

The 16S amplicon sequencing data, starting from raw reads, underwent processing into taxon density tables through the utilization of the LotuS2 pipeline (Less OTU Scripts 2). Key steps in this process included demultiplexing, quality filtering, and dereplication of reads, which were carried out with the assistance of a straightforward demultiplexer (sdm). Additionally, chimeric sequences were identified and eliminated using UCHIME algorithms. Taxonomic postprocessing and sequence clustering, employing combined databases such as SILVA, GG2, and HITdb, were performed using LCA and DADA2 sequence clustering algorithms, respectively. A total of 41,047,536 raw reads were processed, of which 34,131,681 (83%) passed quality filtering and were retained for downstream analyses. These details, including package versions and filtering thresholds, have been added to the Methods section to enhance reproducibility. The total number of reads accounted for 28,147,916 employing a similarity threshold of 97% for distance comparison. After filtering, the sequences were categorized into 7754 ASVs and were attributed to the bacterial domain, 25,637,043 reads remained in the matrix. Analysis of metabolic pathways. To further investigate the functional potential of the microbial community, metabolic pathway analysis was performed using PICRUSt2 (Phylogenetic Investigation of Communities by Reconstruction of Unobserved States) version 2.5.1. Following default settings, PICRUSt2 was used to predict functional metagenomic profiles based on 16S rRNA gene data. This approach provides a *predictive (inferred)* estimation of microbial functional potential rather than direct metagenomic measurement).

### Statistical analysis

Data analysis was performed using Python 3.12. The data was rarefied, and relative abundance was calculated using total sum scaling (TSS). Biodiversity metrics were calculated using the scikit-bio v0.6.3 library. Alpha diversity richness was assessed using Observed, Chao1, and ACE indexes, evenness using the Shannon, Pielou, and Simpson indexes, and phylogenetic diversity using the Faith index. Beta diversity was assessed using unweighted and weighted metrics, namely Jaccard, unweighted UniFrac (U-UniFrac), Bray–Curtis, and weighted UniFrac (W-UniFrac). Significance of grouping was assessed using PERMANOVA, ANOSIM tests with 999 permutations. PERMDISP was used to test for homogeneity of multivariate dispersions. Differential analysis for taxonomic and functional profiles was performed using unpaired t-tests or Mann–Whitney U-tests, where appropriate, with 95% CI calculation for difference between means (non-overlapping 95%CI & p ≤ 0.05) and using LEfSe analysis (LDA > 2 & p ≤ 0.05). Correlation analysis between irradiation dose and species abundance was performed using Pearson’s correlation coefficient with FDR correction; the significance threshold for adjusted values was set at 0.05. Statistical comparisons were performed using NumPy v2.0.1, SciPy v1.15.1, and statsmodels v0.14.4 packages. Visualization was created using the Matplotlib v3.10.0 and seaborn v0.13.2 libraries.

## Results

### Characteristics of experimental animals

Body weight dynamics were monitored across all experimental groups throughout the study period (Table 1). While all groups exhibited a modest increase in mean body weight at 72 h post-intervention, no statistically significant differences (p > 0.05) were observed between the groups.

**Table 1:** Weight of experimental animals before and after the experiment (after 72 h)

Parameters	Cold (^30^SiO_2_) group, n = 5	Hot (^31^SiO_2_) group, n = 5	Gamma group (Gamma), n = 5	Control (Cntrl), n = 5
*Weight of rats before experiment*				
Weight, M ± Sd	298.2 ± 60.6	303.2 ± 57.5	283.4 ± 60.5	271.2 ± 68
*Weight of rats after experiment*				
Weight, M ± Sd	297 ± 49.5	308.9 ± 57.4	287.8 ± 57.6	277.2 ± 64.4
P-value	0.91^a^	0.70^a^	0.44^a^	0.48^a^

^a^Paired T-test

Outbred rats with Specific-pathogen-free (SPF) status were exposed to various external influences: radioactive silicon dioxide ^31^SiO_2_ microparticles, non-radioactive silicon dioxide ^30^SiO_2_ microparticles, gamma radiation and control group. The body weight of the animals in all groups increased 72 h after the experiment, and there were no significant changes in weight between the groups.

### Compositional analysis of gut microbiota after 90 min

**Fig. 2:**
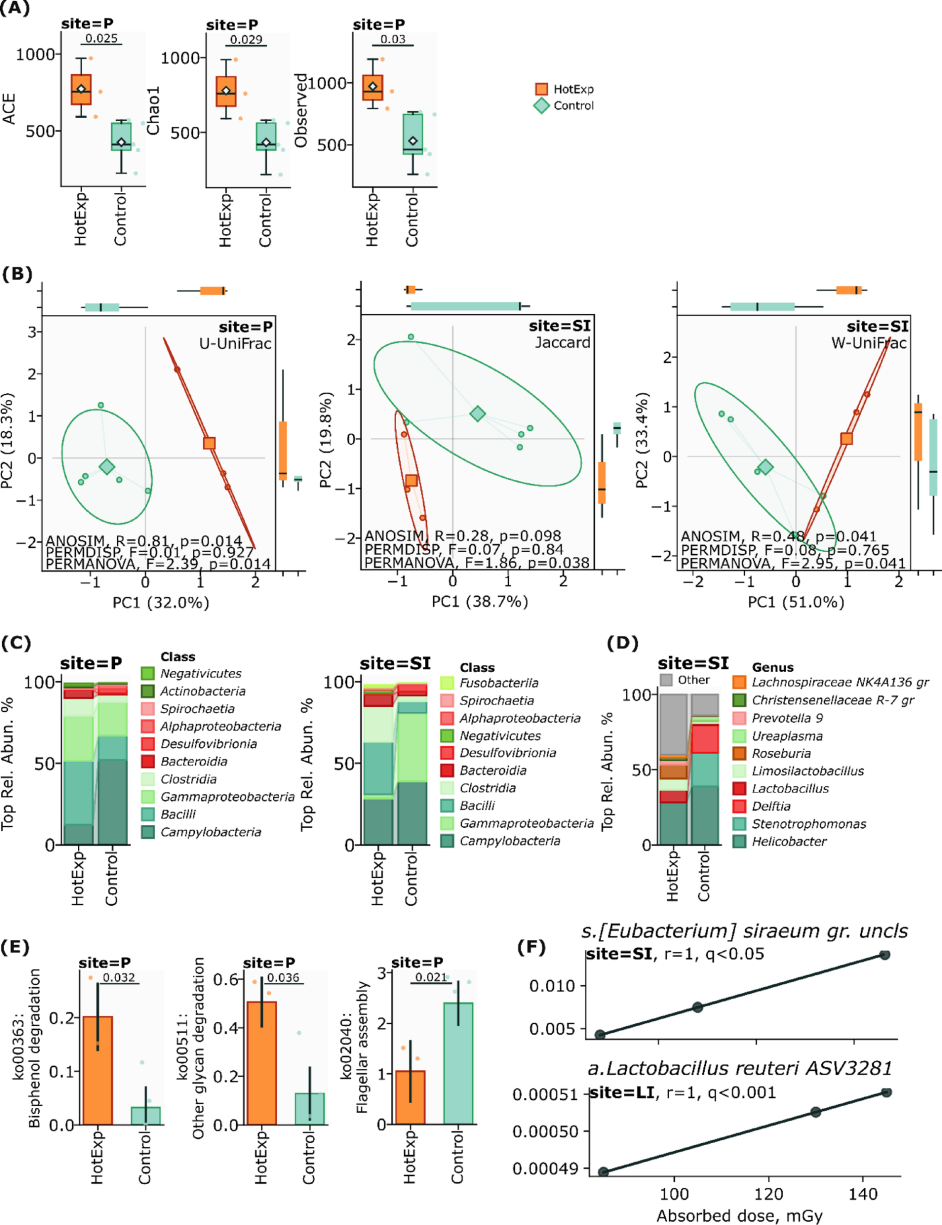
**A** alpha diversity in Peyer’s patches (site—P) after 90 min radioactive ^31^SiO_2_ exposure Hot (^31^SiO_2_) versus control group, ACE (p = 0.025), Chao1 (p = 0.029), Observed (p = 0.03). **B** Beta diversity in small intestine (site—SI) and in Peyer’s patches (site—P) after 90 min radioactive ^31^SiO_2_ exposure Hot (^31^SiO_2_) versus control group, W-Unifrac (PERMANOVA, F = 2.95, p = 0.041), (PERMDISP, F = 0.08, p = 0.765), (ANOSIM, R = 0.48, p = 0.041), Jaccard (PERMANOVA, F = 1.86, p = 0.038), (PERMDISP, F = 0.07, p = 0.84), (ANOSIM, R = 0.28, p = 0.098) peyer’s patches (Site—P) U-Unifrac (PERMANOVA, F = 2.39, p = 0.014), (PERMDISP, F = 0.01, p = 0.927), (ANOSIM, R = 0.81, p = 0.014). **C** The average relative abundance of taxa in the Peyer’s patches and small intestine on the class and genus levels (site—P) and (Site = SI), **D** the average relative abundance of taxa in the small intestine (Site—SI) on the genus level. **E** Metabolic pathways in Peyer’s patches (site—P) after 90 min of exposure. **F** Correlation between absorbed doses of internal irradiation by ^31^SiO_2_ microparticles and: (a) specific bacterial taxa of *a*_*Lactobacillus_reuteri_ASV3281* in the large intestine (LI); (b) specific bacterial taxa of *s_Eubacterium_siraeum_group* in the small intestine (SI).

Observations in 90 min after exposure revealed changes in alpha and beta diversity across all investigated ecological niches, although most changes did not achieve statistical significance (p ≥ 0.05). However, we determined the significant modifications in alpha diversity indices, specifically ACE (p = 0,025), Chao1 (p = 0,029), and Observed (p = 0,03) in Peyer’s patches (Fig. 2A), suggesting a distinct response pattern in these immunological structures.

The observed shifts in community composition were detected in small intestine using Jaccard (PERMANOVA, F = 1.86, p = 0.038), (PERMDISP, F = 0.07, p = 0.84), (ANOSIM, R = 0.28, p = 0.098), W-UniFrac (PERMANOVA, F = 2.95, p = 0.041), (PERMDISP, F = 0.08, p = 0.765), distances and in Peyer’s patches using U-UniFrac (PERMANOVA, F = 2.39, p = 0.014), (PERMDISP, F = 0.01, p = 0.927), (ANOSIM, R = 0.81, p = 0.014) distance (Fig. 2B), providing evidence for radiation-induced perturbations in the gut microbiota phylogenetic compartment. These findings demonstrate that ionizing radiation from beta particles may rapidly induce changes in gut microbial ecology, with particularly pronounced effects in immunologically active sites such as Peyer’s patches.

Exposure to radioactive silicon dioxide ^31^SiO_2_ microparticles induced distinct alterations in the microbial communities across different intestinal tissues, with tissue-specific patterns of restructuring observed in the small intestine, Peyer’s patches, and large intestine. Analysis of the taxonomic shifts revealed complex responses at multiple hierarchical levels, from phylum to species, reflecting the specialized physiological roles and microenvironments of each tissue type.

In the small intestine, exposure to radioactive silicon dioxide ^31^SiO_2_ microparticles led to substantial shifts in major phyla, characterized by a dramatic increase in Firmicutes (10.77% to 56.16%, p = 0.03) and a corresponding decrease in Proteobacteria (42.58% to 3.05%, p = 0.09). Campylobacterota exhibited a moderate reduction from 39.04% to 28.50%, (p = 0.72) (Supplementary Fig. 1). At the class level, significant changes included marked increases in both *Bacilli* (7.06% to 32.10%, p = 0.11) and *Clostridia* (3.54% to 22.03%, p = 0.17), while *Gammaproteobacteria* showed a substantial decrease (41.90% to 2.54%, p = 0.09) (Fig. 2C).

Peyer’s patches demonstrated distinct response patterns, reflecting their specialized immunological microenvironment. The most notable change was a dramatic decrease on phylum level in Campylobacterota (52.12% to 12.29%, p = 0.10), accompanied by considerable increases in Firmicutes (19.66% to 51.69%, p = 0.17) and Proteobacteria (22.97% to 27.95%, p = 0.79) (Supplementary Fig. 2). Class-level analysis revealed substantial increases in both *Bacilli* (14.87% to 39.18%, p = 0.25) and *Gammaproteobacteria* (20.58% to 27.74%, p = 1.0), suggesting adaptation to the altered immunological landscape (Fig. 2C).

Analysis at the genus level revealed tissue-specific patterns of microbial community restructuring. In the small intestine, *Helicobacter* abundance decreased moderately (38.91% to 28.29%, p = 0.72), while *Roseburia* showed a visible increase (0.17% to 9.21%, p = 0.14) (Fig. 2D). Peyer’s patches exhibited more pronounced changes, with *Helicobacter* showing a dramatic decrease (52.00% to 12.07%, p = 0.10) and notable increases in both *Stenotrophomonas* (14.04% to 19.60%, p = 0.4) and *Lactobacillus* (0.73% to 14.54%, p = 0.07) (Supplementary Table 1).

Species-level analysis further highlighted the tissue-specific nature of the response to radiation exposure. In the small intestine, *Stenotrophomonas maltophilia* was nearly eliminated (16.25% to 0.001%, p = 0.16), while *Lactobacillus reuteri* showed a moderate increase (1.74% to 6.64%, p = 0.25). Conversely, Peyer’s patches demonstrated an increase in *S. maltophilia* (8.75% to 17.59%, p = 0.57) and a more pronounced elevation in *L. reuteri* abundance (2.02% to 10.79%, p = 0.14) (Supplementary Table 2).

Small intestine sample analysis revealed significant alterations at the family level, with marked decreases in *Xanthomonadaceae* (22.35% to 0.001%, p = 0.08) and *Comamonadaceae* (18.38% to 0.014%, p = 0.09), contrasted by substantial increases in *Lactobacillaceae* (3.88% to 23.15%, p = 0.19) and *Lachnospiraceae* (1.11% to 16.80%, p = 0.07). These changes were accompanied by the near-complete elimination of certain species, including *S. maltophilia* and *Ureaplasma* gut metagenome (Supplementary Table 3).

The effect of radioactive silicon dioxide ^31^SiO_2_ microparticles significantly affected the change in predicted metabolic pathways in Peyer’s patches compared with the control group, which was not exposed to any external influences. Exposure to radioactive silicon dioxide ^31^SiO_2_ microparticles significantly increased the degradation activity of bisphenol (p = 0.032) and the degradation of glycans (p = 0.036). Interestingly, after exposure to radioactive silicon dioxide ^31^SiO_2_ microparticles, the metabolic pathway of the Flagellar assembly (p = 0.021) remains significantly lower compared to the control group (Fig. 2E).

### Absorbed doses correlation with bacterial taxa in gut microbiota after 90 min of hot ^31^SiO_2_ microparticles exposure

Correlation analysis of the absorbed doses 90 min after irradiation in rat organs revealed correlation between exposure to hot ^31^SiO_2_ and specific bacterial taxa in the large (Site—LI) and small (Site—SI) intestines with internal absorbed doses ranging from 0 to 5.3 mGy in the small intestine and from 0 to 145 mGy in the large intestine. We have identified two bacterial taxa that show a positive correlation with absorbed doses (q < 0.05, FDR) resulting from internal exposure to by ^31^SiO_2_ microparticles. We observed the correlation between absorbed doses with specific bacterial taxa of *a*_*Lactobacillus_reuteri_ASV3281* in the large intestine (r = 1, q < 0.001) and specific bacterial taxa of *s_Eubacterium_siraeum_group* in the small intestine (r = 1, q < 0.05) (Fig. 2F).

The observed correlations between absorbed doses of internal irradiation under the influence of hot ^31^SiO_2_ microparticles and the composition of the gut microbiota in the large and small intestines indicate a pronounced restructuring of bacterial communities in response to internal radiation exposure by radioactive microparticles. These results highlight the selective effect of internal irradiation by radioactive microparticles on microbial communities, where *Lactobacillus_reuteri_ASV3281* and *s_Eubacterium_siraeum_group* exhibits correlations which may play a key role in adaptation to radiation stress. These patterns indicate that exposure to hot ^31^SiO_2_ microparticles can lead to a restructuring of the gut microbiota, favoring radiation-resistant taxa and suppressing key commensal species.

### Microbiome shifts after 72 h

#### Small intestine and Peyer patches’ microbiota

**Fig. 3:**
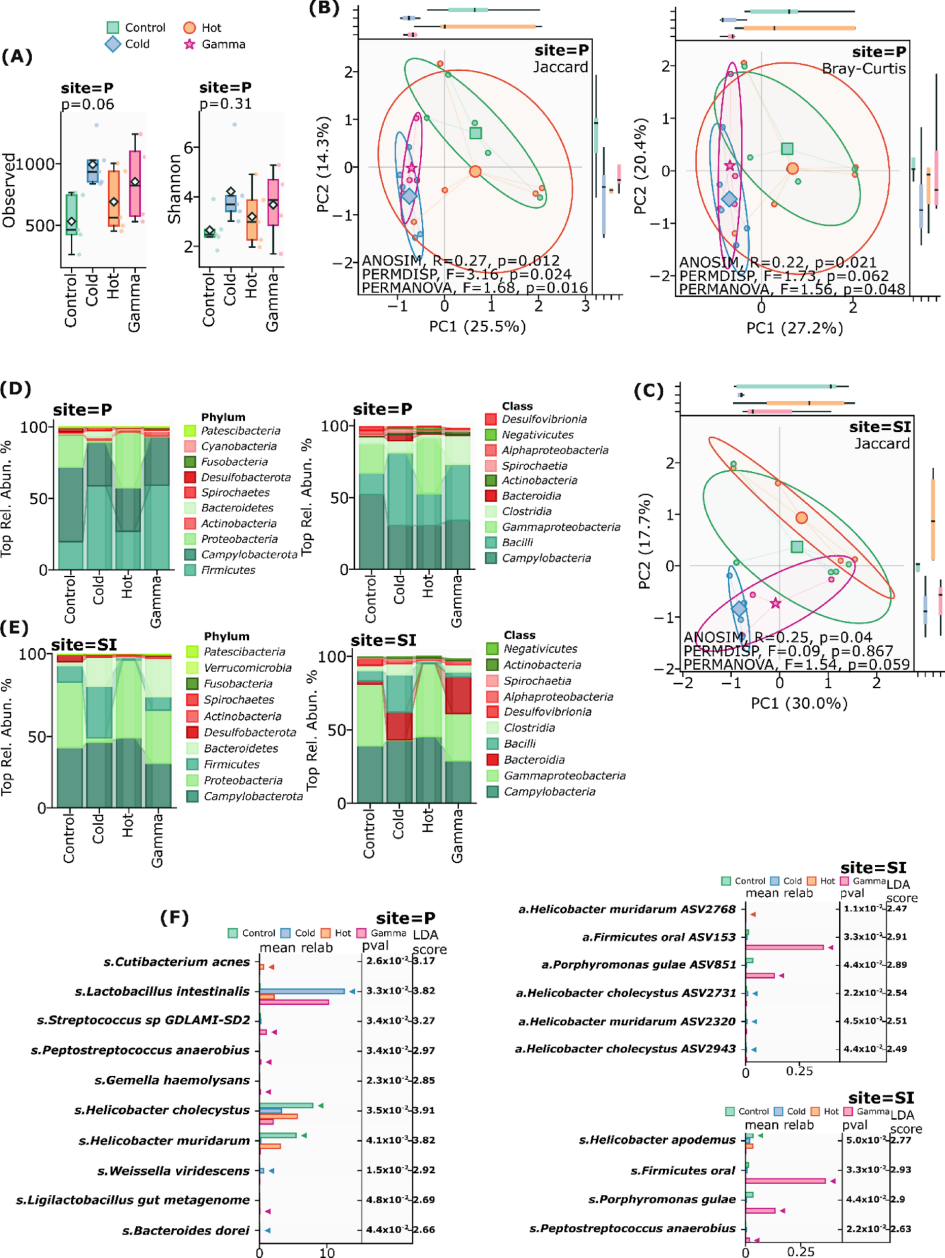
**A** Alpha diversity in Peyer’s patches (Site—P), after 72 h. Observed (p = 0.06), Shannon (p = 0.31). **B** Beta diversity of Peyer’s patches (Site—P), Bray–Curtis (ANOSIM, R = 0.22; p = 0.021; PERMDISP, F = 1.73, p = 0.062; PERMANOVA, F = 1.56, p = 0.048), Jaccard (ANOSIM, R = 0.27; p = 0.012; PERMDISP, F = 3.16, p = 0.024; PERMANOVA, F = 1.68, p = 0.016). **C** Beta diversity of small intestine (Site—SI), Jaccard (ANOSIM, R = 0.25; p = 0.04; PERMDISP, F = 0.09, p = 0.867; PERMANOVA, F = 1.54, p = 0.059). **D** Relative abundance of taxa on phylum and class levels in Peyer’s patches (Site—P). **E** Relative abundance of taxa on phylum and class levels in small intestine (Site—SI). **F** Significant differences in taxonomic composition between the experimental groups in Peyer’s patches (Site—P) after 72 h of exposure. **G** Significant differences in taxonomic composition between the experimental groups in small intestine (Site—SI) after 72 h of exposure

Investigation of ^31^SiO_2_-induced alterations in the gut microbiota revealed compartment-specific responses at 72 h post-exposure. In the small intestine, ^31^SiO_2_ exposure led to a reduction in alpha diversity metrics (observed features, Shannon index, Simpson diversity, Pielou evenness, and Faith’s phylogenetic diversity), although these changes did not achieve statistical significance (p ≥ 0.05). Remarkably, Peyer’s patches demonstrated a contrasting pattern, exhibiting elevated alpha diversity following ^31^SiO_2_ exposure, though these alterations also remained non-significant (Fig. 3A).

Beta diversity analyses demonstrated significant restructuring of microbial communities across intestinal compartments. The small intestine exhibited substantial community reorganization, evidenced by significant clustering patterns using Jaccard distance (ANOSIM, R = 0.25; p = 0.04; PERMDISP, F = 0.09, p = 0.867; PERMANOVA, F = 1.54, p = 0.059) (Fig. 3C). Peyer’s patches displayed more pronounced compositional shifts, with significant differentiation across multiple distance metrics: Bray–Curtis (ANOSIM, R = 0.22; p = 0.021; PERMDISP, F = 1.73, p = 0.062; PERMANOVA, F = 1.56, p = 0.048), Jaccard (ANOSIM, R = 0.27; p = 0.012; PERMDISP, F = 3.16, p = 0.024; PERMANOVA, F = 1.68, p = 0.016) (Fig. 3B).

Taxonomic profiling revealed compartment-specific alterations in microbial composition following ^31^SiO_2_ radioactive exposure. Compared to control, in the small intestine, we observed a five-fold reduction of Firmicutes (10.77% to 2.10%, p = 0.28) accompanied by moderate increase in Proteobacteria (42.58% to 50.50%, p = 0.79) and Campylobacterota (39.04% to 45.44%, p = 0.83) on phylum level. Class-level analysis revealed substantial decreases in *Bacilli* (7.06% to 1.52%, p = 0.11) and *Clostridia* (3.54% to 0.549%, p = 0.50), concurrent with increase in *Gammaproteobacteria* (41.90% to 49.60%, p = 0.80) (Fig. 3E).

Peyer’s patches exhibited distinct microbial dynamics on the phylum level, characterized by reduction in Campylobacterota (52.12% to 30.42%, p = 0.55) and concurrent increases in Firmicutes (19.66% to 26.96%, p = 0.70) and Proteobacteria (22.97% to 39.26%, p = 1.0). Notable class-level alterations included expansions in both *Bacilli* (14.87% to 22.38%, p = 1.0) and *Gammaproteobacteria* (20.58% to 38.96%, p = 1.0) (Fig. 3D).

Using Linear discriminant analysis Effect Size (LEfSe) analysis, we identified distinct taxonomic signatures associated with each type of exposure at both sites.

The mucosal-associated microbiota of small intestine showed a highly specific response to hot exposure, with enrichment limited to a single taxonomic lineage (*Helicobacter muridarum ASV2768*, LDA = 2.47, p = 0.011), while gamma radiation induced broader changes affecting three distinct bacterial lineages, including *Firmicutes oral* taxon (LDA = 2.93, p = 0.033), *Porphyromonas gulae* (LDA = 2.90, p = 0.043), and *Peptostreptococcus anaerobius* (LDA = 2.63, p = 0.022) (Fig. 3G). In contrast, the Peyer’s patch microbiota exhibited more diverse responses across all treatments. Hot exposure specifically enriched *Cutibacterium acnes* (LDA = 3.17, p = 0.025), while gamma radiation induced significant increases in multiple species, including *Lactobacillus intestinalis* (LDA = 3.82, p = 0.033), *Streptococcus sp.* GDLAMI-SD2 (LDA = 3.27, p = 0.034), and *Gemella haemolysans* (LDA = 2.85, p = 0.22). Notably, cold exposure, which was not significant in mucosal communities, induced specific changes in Peyer’s patch microbiota, including enrichment of *Weissella viridescens* (LDA = 2.92, p = 0.014) and *Bacteroides dorei* (LDA = 2.66, p = 0.04) (Fig. 3F).

Comparative analysis revealed distinct microbial signatures between the two sites, with Peyer’s patches showing higher taxonomic diversity and higher LDA scores compared to mucosal communities. The control group maintained specific enrichment of Helicobacter species in Peyer’s patches, suggesting a potential role in immune system homeostasis.

### Mucosal-associated microbiota of the large intestine (MAM) and luminal microbiota (LM)

**Fig. 4:**
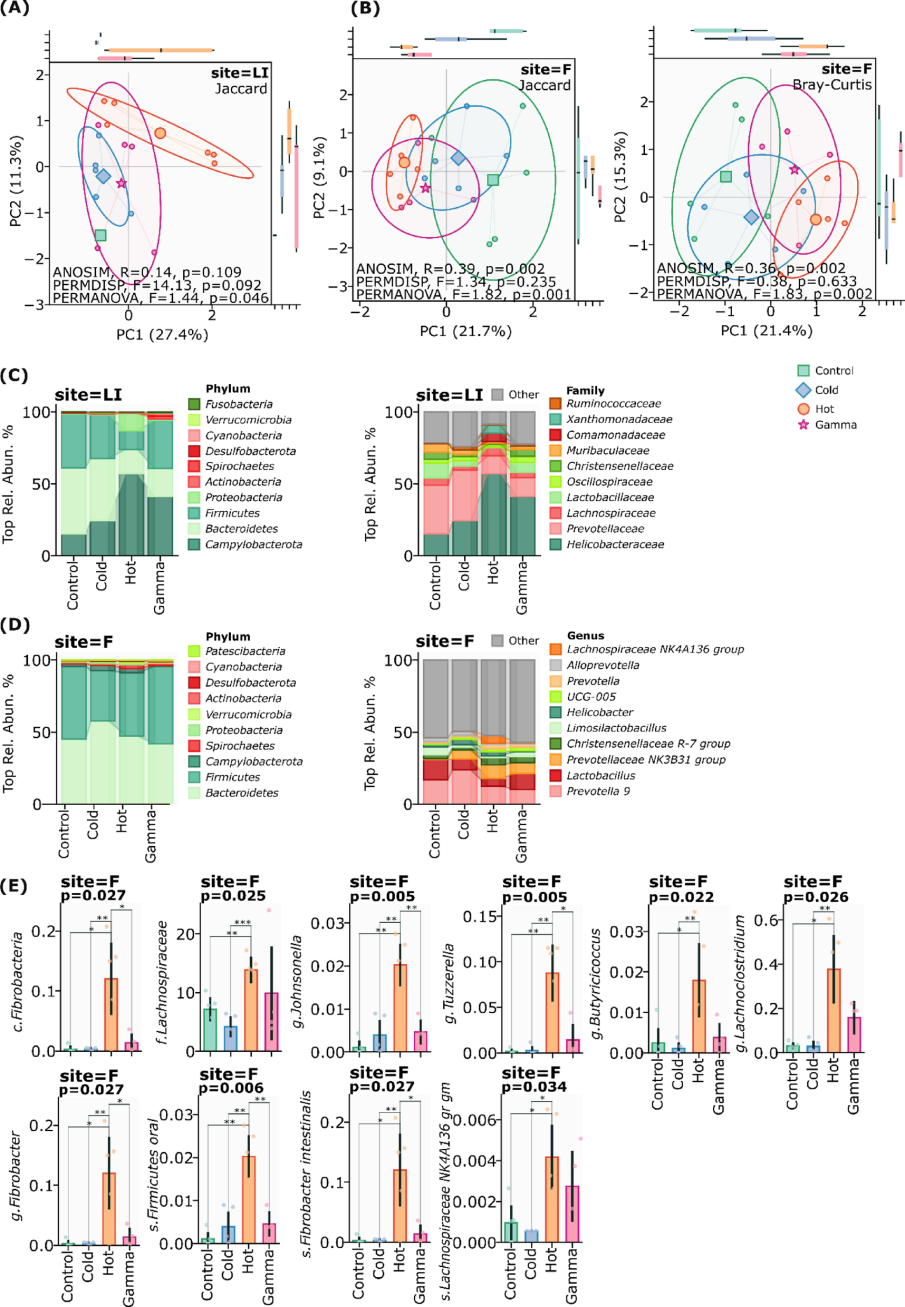
**A** Beta diversity of large intestine (Site—LI), Jaccard (PERMANOVA, F = 1.44; p = 0.046; PERMDISP, F = 14.13, p = 0.092; ANOSIM, R = 0.14, p = 0.109). **B** Beta diversity of fecal samples (Site—F), Bray–Curtis (ANOSIM, R = 0.36; p = 0.002; PERMDISP, F = 0.38, p = 0.633; PERMANOVA, F = 1.44; p = 0.046), Jaccard (ANOSIM, R = 0.39; p = 0.002; PERMDISP, F = 1.34, p = 0.235; PERMANOVA, F = 1.82, P = 0.001). **C** The relative abundance of taxa on phylum and family levels in large intestine. **D** Relative abundance of taxa on phylum and genus levels in fecal samples. **E** Relative abundance of taxa on various levels in fecal samples which significantly increased in hot group (site—F)

At 72 h post-exposure to radioactive ^31^SiO_2_, we observed distinct compartment-specific responses in the large intestinal microbiota. The mucosal-associated microbiota (MAM) exhibited decreased alpha diversity, while the luminal microbiota demonstrated elevated diversity across all ecological indices (Observed features, Shannon index, Simpson diversity, Pielou evenness, and Faith’s phylogenetic diversity), although these changes did not achieve statistical significance (p ≥ 0.05). Beta diversity analyses revealed significant community restructuring in both compartments. The MAM showed significant compositional shifts using Jaccard distance metrics (PERMANOVA, F = 1.44; p = 0.046; PERMDISP, F = 14.13, p = 0.092; ANOSIM, R = 0.14, p = 0.109) (Fig. 4A), indicating clear differentiation between ^31^SiO_2_-exposed and control groups. Notably, the luminal microbiota demonstrated even more pronounced restructuring, with significant clustering patterns across multiple ecological distance metrics: Bray–Curtis (ANOSIM, R = 0.36; p = 0.002; PERMDISP, F = 0.38, p = 0.633; PERMANOVA, F = 1.44; p = 0.046), Jaccard (R = 0.39; p = 0.002; PERMDISP, F = 1.34, p = 0.235; PERMANOVA, F = 1.82, P = 0.001) (Fig. 4B).

Taxonomic profiling revealed distinct microbial architectures and exposure-specific alterations in intestinal compartments. ^31^SiO_2_ exposure led to substantial microbial community reorganization in MAM, notably increasing Proteobacteria (0.35% vs 12.08%, p = 0.71 to control group) and Campylobacterota (15.28% vs 57.1%, p = 0.34 in control group) in the large intestine on phylum level (Fig. 4C). At the family level, *Xanthomonadaceae* (0.001% vs 5.6%, p = 0.72 in control group) and *Comamonadaceae* (0,001% vs 5.85%, p = 0.72 in control group) showed marked elevations (Fig. 4C), primarily driven by *Stenotrophomonas_maltophilia* expansion on species level (0.001% to 4.17%, p = 0.72 in control group). In contrast, the LM remained relatively stable at phylum level, with minor shifts in Bacteroidetes (47.32%, p = 0.83) and Firmicutes proportions (43.8%, p = 0.53) compared to control values (45.3% and 50.3%, respectively) (Fig. 4D). However, genus-level changes were observed in *Prevotellaceae_NK3B31_group* (9.4% vs. 0.7% in control, p = 0.007, non-overlapping 95CI%) and *Lachnospiraceae_NK4A136_group* (5.4% vs. 0.2% in control, p = 0.008, non-overlapping 95CI%) (Fig. 4D).

The number of *Prevotellaceae_NK3B31_group* bacteria increased in all the studied groups compared to the control group (p = 0.01). Interestingly, the relative number of *Lactobacillus* bacteria decreased substantially (p = 0.45) after exposure to radioactive (Hot, p = 0.24) and non-radioactive (Cold, p = 0.38) silicon dioxide. It is worth noting that in the group with radioactive silicon dioxide, there was a significant increase in bacteria of the genus *Lachnospiraceaea_NK4A136_group* (5.4%), while in the other groups their number did not increase significantly.

Exposure to radioactive silicon dioxide ^31^SiO_2_ microparticles significantly modulated the intestinal microbial community structure, as evidenced by distinct taxonomic shifts compared to control conditions. Differential analysis revealed bidirectional changes in bacterial populations with potential implications for host-microbe interactions.

Several bacterial taxa demonstrated significant enrichment following radioactive silicon dioxide ^31^SiO_2_ exposure. *Fibrobacter intestinalis* and related *Fibrobacter* species showed marked increase in abundance (p = 0.027). This enrichment of the *Fibrobacteria* class (p = 0.027), suggesting enhanced capacity for plant polysaccharide degradation. Similarly, *Lachnospiraceae_NK4A136* demonstrated significant enrichment (p = 0.034), potentially indicating adaptation to altered nutrient availability through enhanced fiber degradation capabilities. The *Lachnospiraceae* family demonstrated significant enrichment (p = 0.025), indicating enhanced short-chain fatty acid (SCFA) production, particularly butyrate and acetate.

Notably, we observed significant elevation in butyrate-producing bacteria, that contribute to intestinal barrier function and immune modulation, particularly *Butyricicoccus* (p = 0.022) and *Lachnoclostridium* (p = 0.026). This enrichment suggests enhanced short-chain fatty acid (SCFA) production capacity, potentially serving as a compensatory mechanism to maintain intestinal epithelial integrity and modulate inflammatory responses. The increased abundance of *Firmicutes oral* (p = 0.006) and *Johnsonella* genus (p = 0.005) indicates potential metabolic versatility, possibly reflecting adaptation to modified environmental conditions through diverse substrate utilization. *Tyzzerella* enrichment (p = 0.005) indicated adaptation in carbohydrate fermentation pathways (Fig. 4E).

These findings reveal sophisticated microbial community adaptations to ^31^SiO_2_ radiation exposure, characterized by enhanced degradative capacity but compromised protective functions. The taxonomic shifts observed suggest potential metabolic reprogramming that may significantly impact host-microbe homeostasis under radiation stress.

**Fig. 5:**
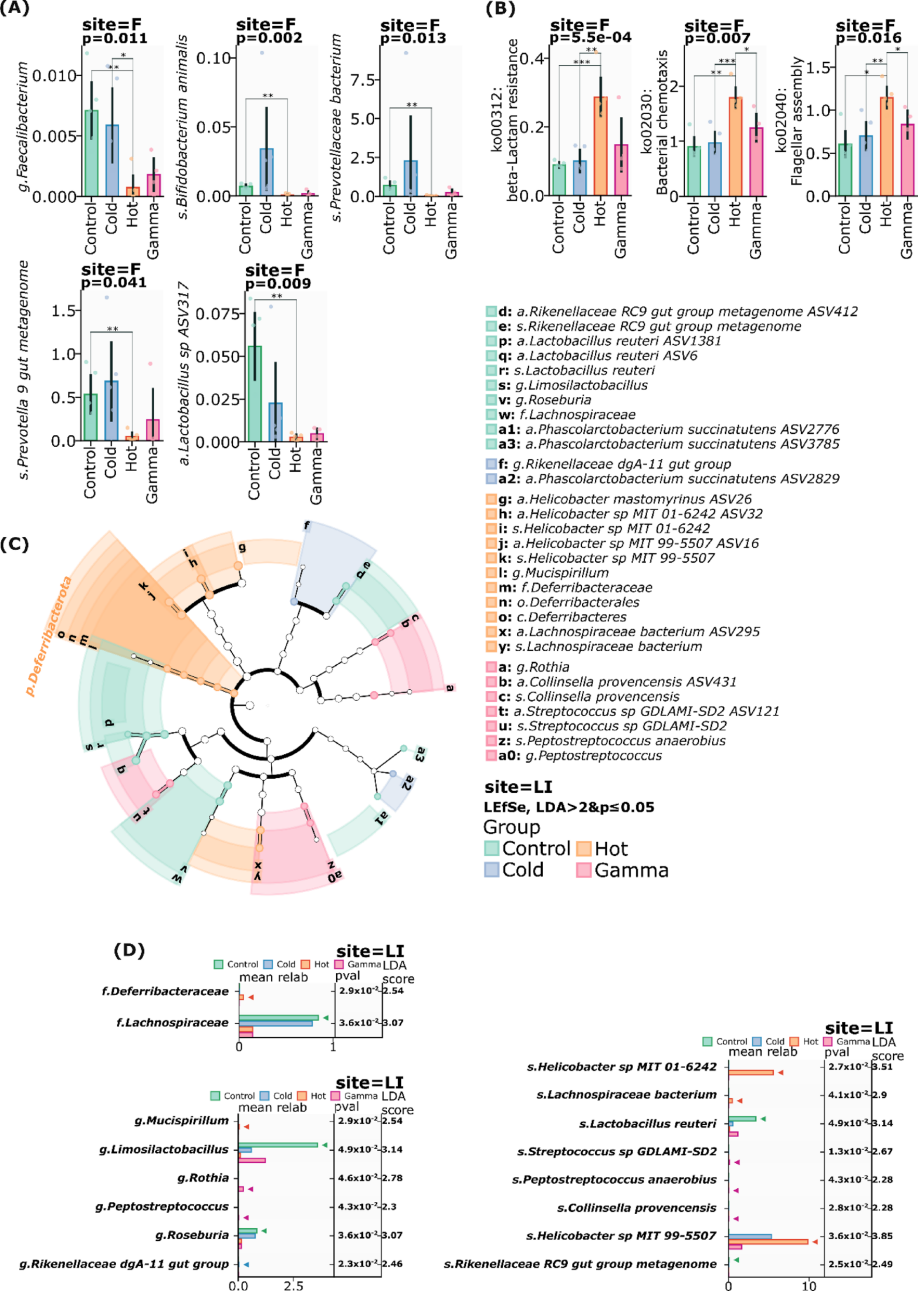
**A** Relative abundance of taxa on different levels in fecal samples which significantly decreased in hot group (site—F). **B** Changes in the metabolic pathways in fecal samples (site—F). **C** LEfSe cladogram of differentially abundant taxa in different groups after exposure in the large intestine (Site—LI). **D** LEfSe analysis of differential bacterial between groups in the large intestine (Site—LI) after exposure

Conversely, the Hot condition ^31^SiO_2_ was characterized by significant reduction in several beneficial bacterial groups. We observed the decreased abundance of *Bifidobacterium animalis* (p = 0.002), potentially affecting lactic acid production and pathogen resistance. Notable reduction in *Faecalibacterium* (p = 0.011) suggested compromised butyrate production and anti-inflammatory functions. The marked reduction in *Prevotella_9_gut* (p = 0.041) and *Prevotellaceae bacterium* (p = 0.013) populations suggests impaired complex carbohydrate metabolism. Most notably, the decreased abundance of *Faecalibacterium* (p = 0.011), known for its anti-inflammatory properties, may predispose the host to enhanced inflammatory responses through reduced immunomodulatory metabolite production (Fig. 5A).

The effect of hot radiation by ^31^SiO_2_ microparticles significantly affected the metabolic pathways in fecal samples compared to the other groups. The potential of resistance to beta-lactam antibiotics has significantly increased (Beta-Lactam resistance, p = 0.0005). Moreover, the radioactive effect of silicon dioxide increases the activation of the flagellar assembly pathway (p = 0.016), and bacterial chemotaxis (p = 0.007) also significantly increased (Fig. 5B).

Intriguingly, the metabolic landscape showed compartment-specific adaptations. The enrichment pattern suggested a shift toward enhanced fiber degradation and complex carbohydrate metabolism, possibly representing a stress response mechanism. This was evidenced by the concurrent increase in cellulose-degrading bacteria (*Fibrobacter*) and reduction in beneficial probiotic species. The enhanced presence of proteolytic (*Lachnoclostridium*) and lipolytic (*Johnsonella*) bacteria further indicated altered nutrient metabolism under Hot exposure conditions.

LEfSe analysis revealed distinct taxonomic signatures Hot ^31^SiO_2_ and Gamma conditions (Fig. 5C), with notable differences between mucosal-associated and luminal microbiota of the large intestine, suggesting temperature-dependent microbial community shifts at the family level. Genus-level analysis unveiled six differentially abundant genera, with distinct patterns across all conditions. *Mucispirillum* was significantly enriched in the Hot condition (LDA = 2.54, p = 2.9e-02), while *Limosilactobacillus* showed strong association with the Control condition (LDA = 3.14, p = 0.0049). Rothia (LDA = 2.78, p = 0.046) and *Peptostreptococcus* (LDA = 2.30, p = 0.043) demonstrated significant enrichment in Gamma group. Notably, *Roseburia* and *Rikenellaceae dgA-11* gut group showed enrichment in Control conditions (LDA = 3.07, p = 0.036; LDA = 2.46, p = 0.023, respectively). At the species level, we identified eight differentially abundant taxa. The Hot condition was characterized by significant enrichment of two *Helicobacter species*: *H. sp. MIT 01–6242* (LDA = 3.51, p = 0.027) and *H. sp. MIT 99–5507* (LDA = 3.85, p = 0.036), along with *Lachnospiraceae bacterium* (LDA = 2.90, p = 0.041). The Control condition exhibited enrichment of *Lactobacillus reuteri* (LDA = 3.14, p = 0.049) and *Rikenellaceae RC9* gut group (LDA = 2.49, p = 0.025). Notably, *Collinsella provencensis* and *Streptococcus sp. GDLAMI-SD2* showed enrichment in Gamma conditions (LDA = 2.28, p = 0.028) and (LDA = 2.67, p = 0.013), while *Peptostreptococcus anaerobius* was enriched in Control (LDA = 2.28, p = 0.043) (Fig. 5D).

## Discussion

Our investigation revealed complex, site-specific responses of the gut microbiota to radioactive silicon dioxide microparticles’ exposure. The observed alterations demonstrated significant gut microbiota structural shifts at both 90 min and 72 h post-exposure, with distinct patterns emerging across different intestinal compartments.

Notably, substantial modifications in alpha diversity indices were observed at 90 min post-exposure, particularly within Peyer’s patches, indicating heightened sensitivity to adverse stimuli in these immunological structures. While these findings align with previous studies demonstrating rapid responsiveness of gut-associated lymphoid tissue to environmental factors, our results uniquely highlight the unprecedented rapidity with which these alterations manifest in the microbial community (Hollingsworth et al. 2021; J. Liu et al. 2021).

The dramatic shifts in major microbial phyla composition at 72 h post-exposure showed a marked reduction of Firmicutes (10.77% to 2.09%) in the small intestine, coupled with a moderate increase in Proteobacteria (42.57% to 50.5%), indicating comprehensive community reorganization. This shift likely represents an adaptive response, given Firmicutes’ metabolic versatility and stress resilience. The rapid alterations in *Bacilli* (7.6% to 32.9%) and *Clostridia* (3.5% to 22.03%) populations within 90 min reflect colonization resistance and metabolic adaptation through bacteriocin, lactic acid, and SCFA production. These findings align with previous research showing dose-dependent changes in microbial composition, with specific genera correlating to radiation intensity and survival outcomes (Jameus et al. 2024; Carbonero et al. 2018). Additionally, we identified significant increase of *Lachnospiraceaea NK4A136 group* on genus level in fecal samples after post radioactive ^31^SiO_2_ exposure, these results are consistent with studies in which the intestines of mice were irradiated with radionuclides yttrium-90 (Y90) carbon microspheres (P. Yang et al. 2025).

The cross-feeding network between *Bacilli* and *Clostridia*, where lactate serves as a substrate, explains the observed synchronized population dynamics. In Peyer’s patches, "Hot" ^31^SiO_2_ exposure led to distinct alterations, characterized by *Campylobacterota* (52.11% to 12.28%) concurrent with *Firmicutes* (19.65% to 51.68%) expansion indicates a specific pattern of microbial community restructuring in response to radioactive silicon dioxide. We identified significant correlations between the absorbed doses in the small and large intestines of rats and specific bacterial taxa ranging up to 5.3 mGy in the small intestine and up to 145 mGy in the large intestine, demonstrating strong positive associations for *a*_*Lactobacillus_reuteri_ASV3281* (r = 1, q < 0.001) and *s_Eubacterium_siraeum_group* (r = 1, q < 0.05). These findings suggest potential biomarkers for monitoring low dose internal radiation exposure. These observations align with previous research showing dose-dependent changes in gut microbiota composition following radiation exposure, including alterations in major phyla like Firmicutes, Bacteroidetes, and Proteobacteria (L. Zhang et al. 2023; Lam et al. 2012*)*. In addition, the unexpected growth of *a_Lactobacillus_reuteri_ASV3281* can be explained by its probiotic properties, such as the production of reuterin, anti-inflammatory effects with IL-10, and the release of IL-22, which mitigate oxidative stress in the small intestine caused by low doses of radiation. These mechanisms are consistent with studies demonstrating that genetically modified *Lactobacillus reuteri* releasing IL-22 (LR-IL-22) promote intestinal radioprotection after irradiation of the entire abdominal cavity, restoring the number of *lactobacilli* and reducing dysbiosis in rodents (Hamade et al. 2022). Integration of metabolomic and 16S rRNA sequencing data has further revealed radiation-induced shifts affecting bacteria and metabolic pathways, particularly in *Lactobacillaceae*, *Lachnospiraceae*, and bile acid metabolism (Goudarzi et al. 2016).

Analysis of metabolic pathways in fecal samples following internal irradiation by ^31^SiO_2_ microparticles revealed significant temporal changes. Initially, at 90 min post-exposure, flagellar assembly pathway activity decreased significantly (p = 0.021), suggesting radiation-induced suppression of bacterial motility. However, by 72 h, flagellar assembly showed significant upregulation (p = 0.016), accompanied by activation of bacterial chemotaxis pathways (p = 0.007), indicating adaptive responses through either motility gene expression recovery or selection of resistant strains. These findings align with previous research demonstrating radiation-induced alterations in bacterial motility and gut microbiota composition, where photocatalysis and low-dose radiation significantly affect flagellar assembly and chemotaxis pathways (J. Zhang et al. 2019; Zhao et al. 2007). Such changes are part of broader effects on gut microbiota, particularly affecting genera like *Clostridium*, *Helicobacter*, and *Bacteroides* (Liu et al. 2019).

Analysis of fecal samples showed increased activation of the bisphenol degradation pathway (p = 0.032) at 90 min post-^31^SiO_2_ irradiation, suggesting enhanced xenobiotic metabolism as part of bacterial stress response. This likely reflects combined physical and radiation stress effects on gut microbiota metabolism. These findings parallel previous research where BPA and gamma radiation showed additive damaging effects on mouse germ cells and steroidogenesis (Wieckowski et al. 2021). Studies have demonstrated that both radiation and BPA independently affect biological systems, with radiation altering gut microbiota composition (Liu et al. 2019; Casero et al. 2017) and BPA impacting reproductive functions (Dobrzyńska and Radzikowska 2013). The early increase in bisphenol degradation followed by flagellar assembly restoration at 72 h suggests a transition from initial stress response to adaptive stability, mirroring patterns observed in mouse reproductive systems under combined stressor exposure (Wieckowski et al. 2021; Casero et al. 2017).

The increased activity of glycan degradation (other glycan degradation, p = 0.036) 90 min after exposure to ^31^SiO_2_ may reflect an attempt by the gut microbiota to use alternative carbon sources under stress conditions. We suggest that local internal exposure to radioactive silicon dioxide ^31^SiO_2_ microparticles could cause local damage to the mucosal layer, increasing the availability of mucin to, for example *Akkermansia Muciniphila* which subsequently increases the activity of other glycan degradation (Derrien et al. 2008).

Several important limitations warrant consideration. As a pilot investigation, while our sample size was sufficient to detect major shifts in microbial communities, it remained relatively modest. The study’s focus on two specific time points (90 min and 72 h post-exposure) may not capture the complete temporal dynamics of gut microbiota alterations. Our analysis, centered on bacterial communities through 16S rRNA sequencing, did not examine other crucial gut microbiota components, including fungi, viruses, and archaea, which may play significant roles in radiation response. The use of a single sex (male rats) and age cohort (10 weeks) limits the generalizability of our findings across different demographic categories. These limitations delineate promising directions for future investigations aimed at expanding our findings and providing more comprehensive understanding of radiation-induced alterations in the gut microbiota.

This investigation presents a comprehensive analysis of radioactive silicon dioxide (^31^SiO_2_) microparticles effects on the gut microbiota, revealing complex and compartment-specific alterations in microbial communities. These changes manifest as significant gut microbiota restructuring, with particularly pronounced modifications in Peyer’s patches, highlighting the sensitivity of immunologically active intestinal structures to radiation exposure. Our findings contribute substantially to understanding the effects induced by internal irradiation in the gut microbiota and establish new avenues for investigating radiation exposure biomarkers.

Our findings extend established temporal patterns of radiation-microbiome interactions. Previous studies demonstrate that 6–24 h constitute a critical dysbiosis window with obligate anaerobe depletion (Kim et al. 2015), while 72 h represents onset of adaptive reorganization with Bacteroides recolonization and incomplete Lactobacillus recovery (Cui et al. 2023); (Guo et al. 2020); (Thandar et al. 2024). However, these patterns derive exclusively from studies of either radiation alone or non-radioactive nanoparticles (Chen et al. 2017)—SiO_2_ NPs at 3 days). No prior work has integrated radioactive nanoparticle exposure with microbiome profiling at any timepoint, creating a fundamental gap in understanding combinatorial nanotoxicity.

Our 90-min data additionally capture ultra-early events absent from existing literature, revealing immediate perturbations preceding the well-characterized 6–24 h dysbiotic cascade. At 3 days, we observed significant microbiome alterations consistent with established radiation patterns, including increased Bacteroidota abundance (30.4% in Ag NP vs. 23.5% in controls), reduced Firmicutes/Bacteroidetes ratio (2.1:1 vs. 2.5:1), and virtual depletion of *Akkermansia muciniphila* (0.04% vs. 1.26%, paralleling Thandar et al. 2024). Nanoparticle-specific effects included increased *Prevotellaceae* (5.6% vs. 1.9%, p = 0.024) in Ag NP-treated rats and significant reductions in probiotic genera *Enterococcus* (0.4% vs. 2.1%, p = 0.038) and *Turicibacter* (0.15% vs. 1.1%, p = 0.008) in SiO_2_ NP group.

The temporal divergence between plasma metabolome perturbations (peak at 14 days) and fecal microbiome shifts (evident at 72 h) suggests compartment-specific recovery kinetics, paralleling (Pannkuk et al. 2019) who demonstrated delayed plasma acylcarnitine normalization despite earlier fecal SCFA restoration in irradiated primates. Notably, persistent depletion of gut microbiota-derived indole-3-acetic acid (IAA) in plasma at 14 days (0.57-fold in Ag NP, p < 0.05) despite partial microbial recovery by 28 days indicates that functional metabolic outputs lag behind taxonomic restoration, with critical implications for understanding prolonged systemic consequences of radiation exposure.

## Conclusion

In conclusion, our investigation reveals that radioactive ^31^SiO_2_ microparticle exposure induces compartment-specific gut microbiota alterations with translational implications for radiation biomonitoring and therapeutic applications.

The rapid temporal dynamics—alpha diversity changes in Peyer’s patches within 90 min and adaptive reorganization at 72 h—combined with dose-dependent taxon correlations (*Lactobacillus reuteri*, *Eubacterium siraeum* group) establish a foundation for developing sensitive biomarkers applicable in radiotherapy monitoring and research settings. The pronounced sensitivity of gut-associated lymphoid tissue highlights the importance of site-specific sampling in radiation exposure assessment protocols.

Clinically, identified dysbiosis patterns offer potential for personalizing radiotherapy protocols through early detection of patients at risk for gastrointestinal toxicity. The radioprotective proliferation of *Lactobacillus reuteri* suggests promising avenues for probiotic interventions in radiation medicine.

## Supplementary Information

Below is the link to the electronic supplementary material.

Supplementary Material 1

## Data Availability

The datasets generated in the present study are available from the corresponding author on reasonable request. The raw 16S sequencing data have been deposited to Zenodo under BioProject ID: 10.5281/zenodo.15181151.
