# Supplementary material for: Multicompartmental analysis of microbiome alterations under radiation stress
Source: AMB Express. 2025 Dec 31;16:9. doi: 10.1186/s13568-025-02002-4 (PMC12864630; doi:10.1186/s13568-025-02002-4)

**Supplementary materials for comment:** PiCRUST2 was utilized; however, only specific pathways are discussed in the manuscript. Could the authors include a supplementary figure showing the top 10–15 pathways affected in each sample group to better visualize the functional changes?

**Supplementary file changes in the metabolic pathways in gut compartments under radiation stress**

[**Changes in the metabolic pathways in Peyer's patches samples (site - P) 1**](#_dlup02vglf4m)

[**Changes in the metabolic pathways in small intestine samples (site - SI) 2**](#_dwno9puzey2o)

[**Changes in the metabolic pathways in large intestine samples (site - LI) 2**](#_l8dz9ve03kpm)

[**Changes in the metabolic pathways in fecal samples (site - F) 3**](#_jo0oqqbohbnw)

#

# Changes in the metabolic pathways in Peyer's patches samples (site - P)


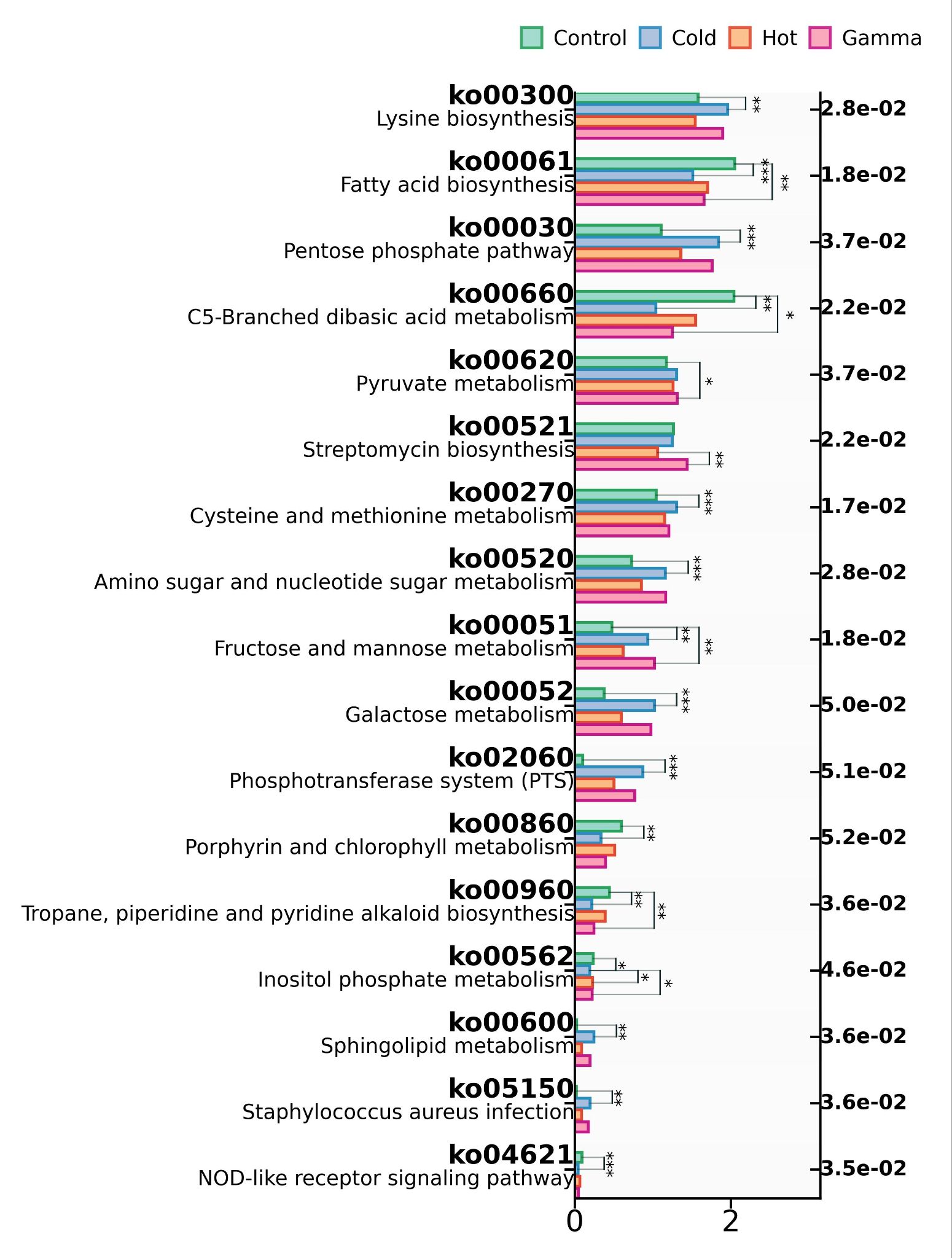


# Changes in the metabolic pathways in small intestine samples (site - SI)


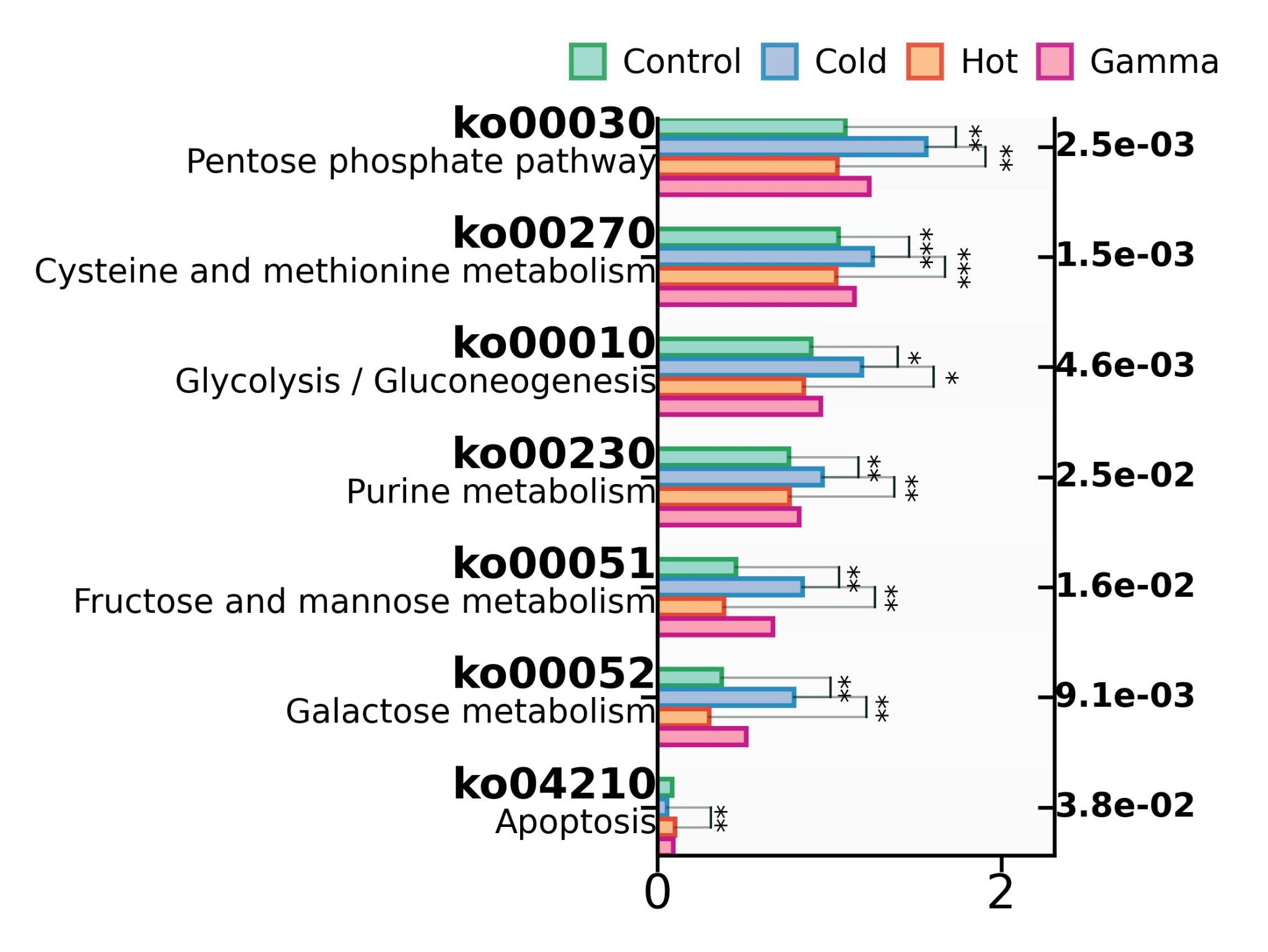


# Changes in the metabolic pathways in large intestine samples (site - LI)


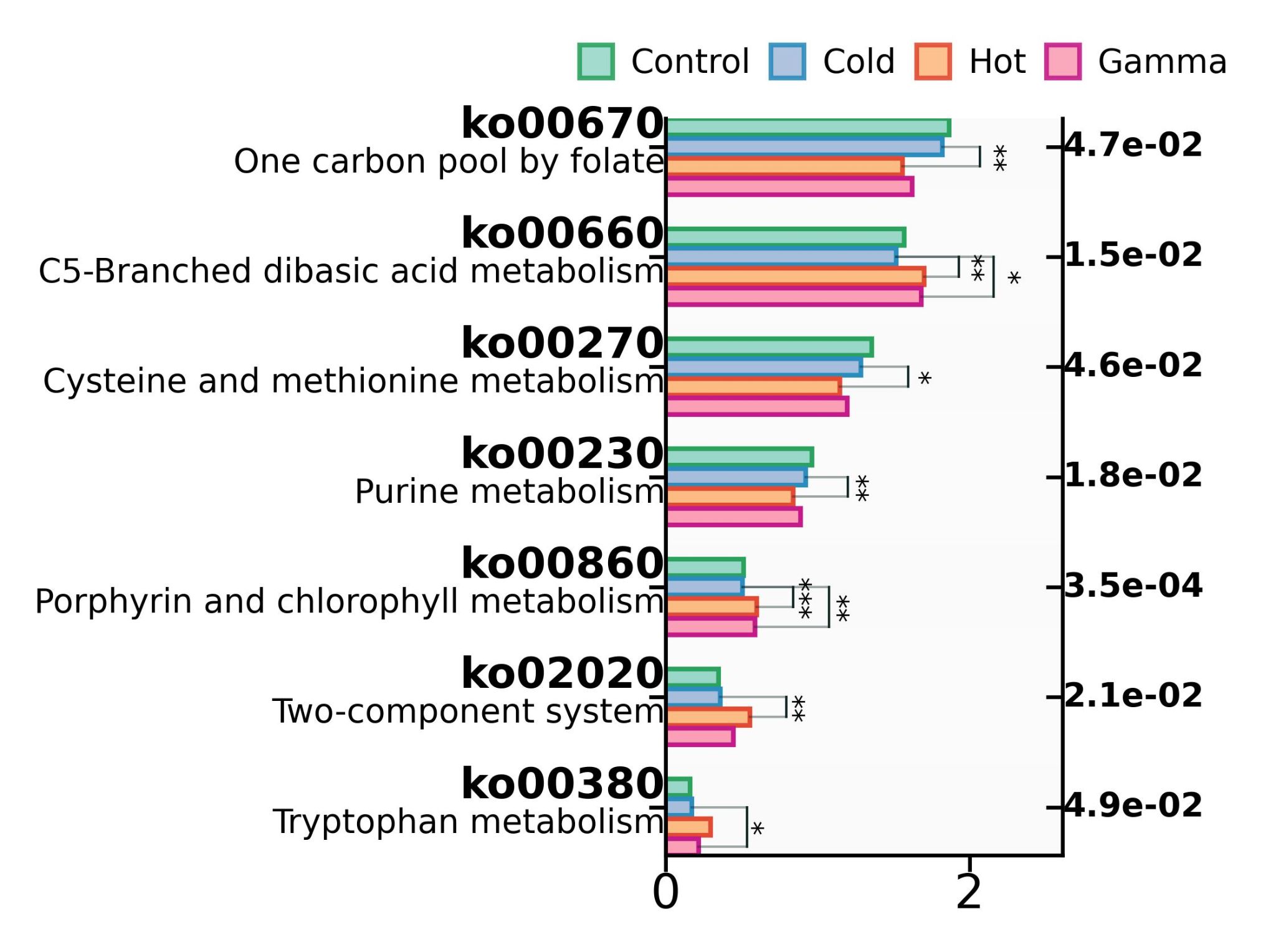


# Changes in the metabolic pathways in fecal samples (site - F)


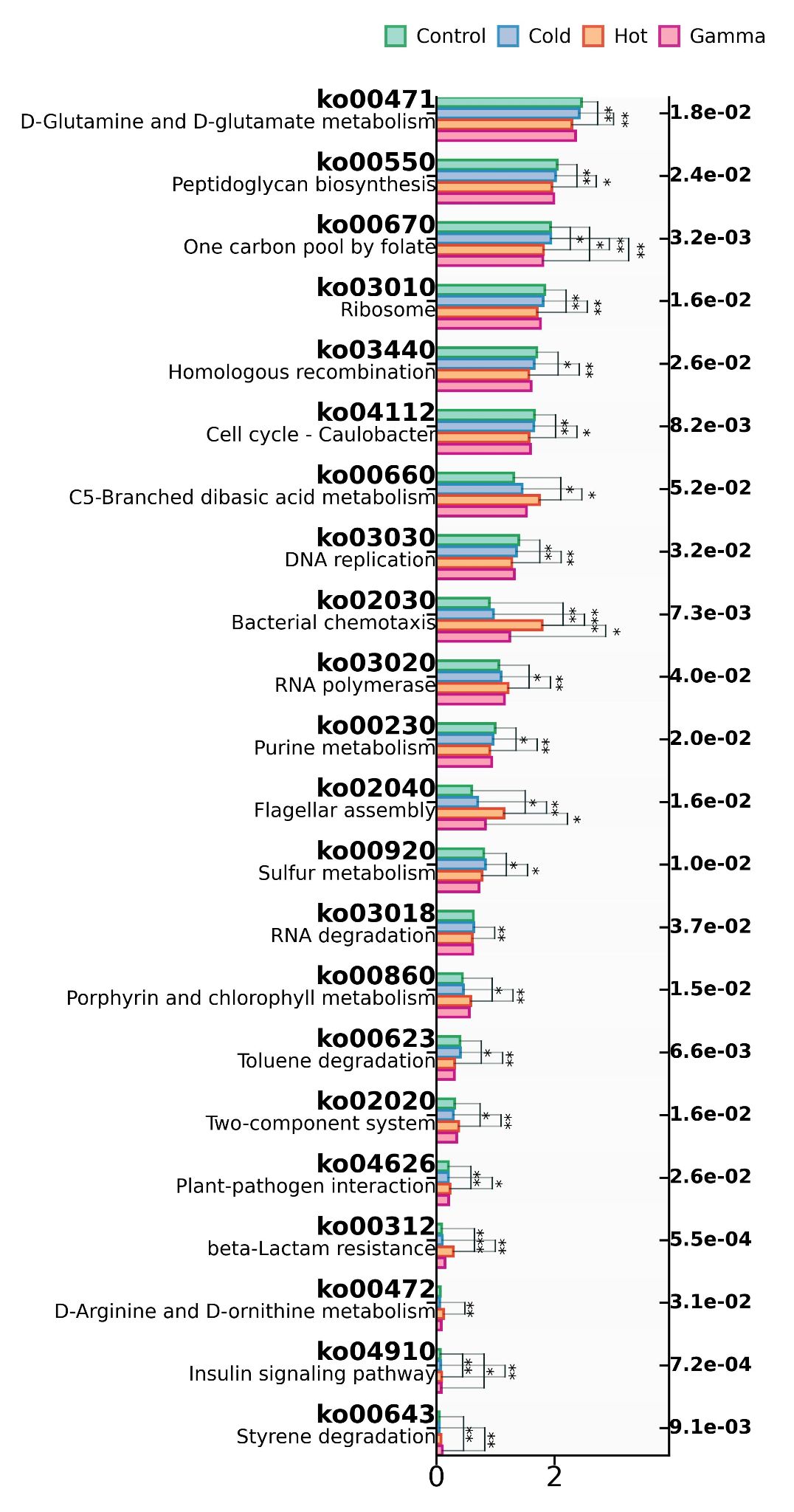

Supplement: Supplementary file 1 — Supplementary Material 1 [file 13568_2025_2002_MOESM1_ESM.docx]
